# Investigation of Gene Expression and DNA Methylation From Seven Different Brain Regions of a Crab-Eating Monkey as Determined by RNA-Seq and Whole-Genome Bisulfite Sequencing

**DOI:** 10.3389/fgene.2019.00694

**Published:** 2019-07-26

**Authors:** Won-Jun Lim, Kyoung Hyoun Kim, Jae-Yoon Kim, Hee-Jin Kim, Mirang Kim, Jong-Lyul Park, Seokjoo Yoon, Jung-Hwa Oh, Jae-Woo Cho, Yong Sung Kim, Namshin Kim

**Affiliations:** ^1^Genome Editing Research Center, Korea Research Institute of Bioscience and Biotechnology (KRIBB), Daejeon, South Korea; ^2^Department of Bioinformatics, KRIBB School of Bioscience, University of Science and Technology (UST), Daejeon, South Korea; ^3^Predictive Toxicity Department, Korea Institute of Toxicology (KIT), Daejeon, South Korea

**Keywords:** whole genome bisulfite sequencing (WGBS), differentially methylated CpG sites, RNA-Seq (quantification) analysis, *Macaca fascicularis*, repeat elements

## Abstract

The crab-eating monkey is widely used in biomedical research for pharmacological experiments. Epigenetic regulation in the brain regions of primates involves complex patterns of DNA methylation. Previous studies of methylated CpG-binding domains using microarray technology or peak identification of sequence reads mostly focused on developmental stages or disease, rather than normal brains. To identify correlations between gene expression and DNA methylation levels that may be related to transcriptional regulation, we generated RNA-seq and whole-genome bisulfite sequencing data from seven different brain regions from a single crab-eating monkey. We identified 92 genes whose expression levels were significantly correlated, positively or negatively, with DNA methylation levels. Among them, 11 genes exhibited brain region-specific characteristics, and their expression patterns were strongly correlated with DNA methylation level. Nine genes (*SLC2A5*, *MCM5*, *DRAM1*, *TTC12*, *DHX40*, *COR01A*, *LRAT*, *FLVCR2*, and *PTER*) had effects on brain and eye function and development, and two (*LHX6* and *MEST*) were previously identified as genes in which DNA methylation levels change significantly in the promoter region and are therefore considered brain epigenetic markers. Furthermore, we characterized DNA methylation of repetitive elements at the whole genome through repeat annotation at single-base resolution. Our results reveal the diverse roles of DNA methylation at single-base resolution throughout the genome and reflect the epigenetic variations in adult brain tissues.

## Introduction

*Macaca fascicularis* (crab-eating macaque) is a non-human primate commonly used for biomedical research aimed at developing drugs for human diseases (Huh et al., 2012). The regions of the nervous system that mediate neuropsychiatric disease are structurally and functionally similar between humans and non-human primates (Ernst and Frisén, 2015). DNA methylation, which generally occurs at cytosines in the context of CpG dinucleotides, is a key epigenetic modification involved in developmental regulation of genes, silencing of gene expression, and cell differentiation (Khavari et al., 2010). Multiple studies have revealed that epigenetic mechanisms are of critical importance in these disorders and have begun to elucidate epigenetic differences between the brains of humans and non-human primates (Zeng et al., 2012). However, in the context of array-based assays for methylation discovery, the resources available for non-human genomes are far less extensive than resources for studies of the human genome. Many previous studies focused on the distribution of DNA methylation, especially in early developmental stages (Li and Zhang, 2014; Li et al., 2016; Luo et al., 2018). Other methylation studies have used antibody-based methods, such as methylated DNA immunoprecipitation (MeDIP) sequencing, which can assess DNA methylation only at methyl-CpG-binding sites (Down et al., 2008; Taiwo et al., 2012; Staunstrup et al., 2016). On the other hand, whole-genome bisulfite sequencing (WGBS) has the significant advantage that it can identify DNA methylation in CpG and non-CpG sites throughout the genome, yielding methylomes with base-pair resolution (Ziller et al., 2015).

However, brain region-specific DNA methylation has not been fully characterized at the genomic level. To address this issue, we performed epigenetic analysis on anatomical regions of the brain, including components of the cerebral cortex, basal ganglia, and limbic system. Because each of these anatomical regions has a different function, diseases involving distinct regions have characteristic symptoms. Each of the seven regions we examined is associated with a particular anatomical location, as well as specific functions and diseases. Dorsolateral prefrontal cortex, which is an area of the prefrontal cortex in humans and non-human primates, is involved in working memory, cognitive flexibility, planning, inhibition, and abstract reasoning (Elliott, 2003). Ventrolateral prefrontal cortex is another part of the prefrontal cortex. Right ventrolateral prefrontal cortex (VLPFC) is thought to play a critical role in motor inhibition (Aron et al., 2004). Angular gyrus, part of the parietal lobe, is involved in number processing, spatial cognition, language, memory retrieval, attention, and theory of mind (Seghier, 2013). Inferior temporal gyrus, in the temporal lobe, is involved in memory and memory recall during identification of objects (Gross, 1992). Caudate nucleus, along with the putamen and *globus pallidus*, is one of three basic structures that constitute the basal ganglia. This region has been implicated in voluntary movement, learning, memory, sleep, and social behavior and is associated with motor processes in Parkinson’s disease (Grahn et al., 2009). Cingulate gyrus, which is part of the limbic system, is involved with emotion formation and processing, learning, and memory; in light of these functions, this region is central to disorders such as depression and schizophrenia (Adams and David, 2007; Drevets et al., 2008). The hippocampus middle is part of the limbic system and plays roles in the consolidation of information from short-term and long-term memories, as well as spatial navigation. In Alzheimer’s disease, the hippocampus is one of the first regions of the brain to suffer damage (Chiu et al., 2004). The study of brain regions associated with neuropsychiatric disorders in non-human primate models has the potential to improve our knowledge and facilitate the translation of research findings to the clinic.

Transcriptome analyses have highlighted gene expression patterns across brain regions (Babak et al., 2015; Labadorf et al., 2015), and several studies have shown that DNA methylation and gene expression are positively correlated in genes that play important roles in brain (Gibbs et al., 2010; Zhang et al., 2010; Chen et al., 2014). However, previous studies of region-specific DNA methylation in brain have assessed only a small percentage of the CpG sites in the genome, and very few studies have compared DNA methylation across multiple brain regions. Moreover, little is known about the methylomic differences between functionally distinct areas of brain regions in normal adults.

Furthermore, approximately half of the mammalian genome consists of DNA repetitive elements (REs), which contribute considerably to the global DNA methylation level (Chalopin et al., 2015), including long terminal repeats (LTRs), long interspersed nuclear elements (LINEs), and short interspersed nuclear elements (SINEs), as well as simple repeats (Papin et al., 2017). Hypo-methylation of transposable repeats such as SINEs and LINEs can increase genome instability, reactivate poorly expressed genes, or disrupt gene function, thereby contributing to disease risk. The level of DNA methylation in REs is a key determinant of their transposition activities, reflected by the fact that over 90% of methylated CpG sites in the human genome occur in REs (Beisel and Paro, 2011). Therefore, given the important contribution of REs to genome-wide CpG content, RE methylation should be investigated throughout the genome.

In this study, we used RNA-seq and WGBS datasets generated from diverse brain regions from *M. fascicularis* to analyze transcriptomic and DNA methylomic diversity, as well as to investigate the relationship between expression patterns and DNA methylation levels. In addition, we performed RE methylation analysis, which can identify regions of the genome characterized by functionally relevant region-specific DNA methylation, and thus represents a unique approach to genomics and neuroscience research.

## Materials and Methods

### Preparation of gDNA and Total RNA

Tissue from seven brain regions (angular gyrus, anterior caudate, cingulate gyrus, hippocampus middle, inferior temporal lobe, dorsolateral prefrontal cortex, and ventrolateral prefrontal cortex) was obtained from a single male crab-eating macaque (*M. fascicularis*). Genomic DNA was isolated using the DNeasy Blood & Tissue Kit (Qiagen, Valencia, CA, USA), and total RNA was isolated using the RNeasy Mini Kit (Qiagen, Hilden, Germany). Concentrations of DNA and RNA were determined using a spectrophotometer, and integrity was monitored by agarose gel electrophoresis. In addition, RNA integrity was monitored on a 2100 Bioanalyzer System (Agilent Technologies, Santa Clara, CA, USA) using the RNA 6000 Nano Kit (Agilent Technologies, Waldbronn, Germany). Samples with an RNA Integrity Number (RIN) > 8 were used for experiments.

### Library Preparation and Construction for Whole-Genome Bisulfite Sequencing and RNA Sequencing

gDNA isolated from brain tissue samples was subjected to bisulfite conversion using the EZ DNA Methylation Gold Kit (Zymo Research, Orange, CA, USA), and then a sequencing library was constructed using the Illumina TruSeq DNA Library Prep Kit (San Diego, CA, USA). Paired-end sequencing was performed on an Illumina HiSeq X Ten sequencing instrument, yielding 150-bp paired-end reads.

RNA sequencing libraries were generated from total RNA using the TruSeq RNA Sample Preparation Kit (Illumina). Each library was diluted to 8 pM and subjected to 76 cycles of paired-read sequencing on an Illumina NextSeq 550 instrument.

### Data Quantification and Processing

The reference sequence (macFas5) was obtained from the UCSC Genome Browser and was used for data processing (http://genome.ucsc.edu). FastQC (v0.11.5) was used to confirm the quality of RNA-seq and WGBS libraries (Andrews, 2010). RNA-seq data generated an average of 144 million 65 × 65-bp paired-end reads, which were aligned to the reference sequence, resulting in an average mapping rate of 68.14% (**Additional file 1: Tables S1**, **S2**). Fragments per kilobase of exon per million mapped fragments (FPKM) was calculated using TopHat2 (v2.0.13) and Cuffdiff (v2.2.1) with default options (Trapnell et al., 2010). A total of 62,752 transcripts were measured using the transcript annotation file from the UCSC Genome Browser.

WGBS data yielded 150 × 150-bp paired-end reads, with an average of 388 million reads per sample, which were trimmed using Trimmomatic (Bolger et al., 2014) (**Additional file 1: Table S3**). DNA methylation levels were quantified using Bismark with default options; reference sequences were converted to map both cytosine and thymine (https://www.bioinformatics.babraham.ac.uk/projects/bismark/). An average of 72.67% of WGBS reads mapped to the converted reference sequences, and sequencing depth of CpG sites averaged 8.19 (**Additional file 1: Table S6**). All sites with depth <10 were discarded, and an average of 16 million sites were ultimately used. Mapped reads were classified as CpG and non-CpG sites and used to calculate DNA methylation levels. Promoter regions were classified as the 2-kb upstream of the transcription start sites (TSSs) using gene annotation files from the UCSC Genome Browser. An average of 16 million sites were calculated by removing sites of read depth less than 10 (**Additional file 1: Table S1**). Annotation of genomic features was performed using the table browser of UCSC Genome Browser. Hyper-methylated and hypo-methylated sites were defined using methylKit (Akalin et al., 2012). To define the intersected regions, BEDTools (v2.25.0-112) was used to calculate the features and DNA methylation levels of the corresponding sites (Quinlan and Hall, 2010).

### Characterization of Brain Regions and Gene Expression Patterns

Gene expression patterns in each region of the monkey brain were examined to characterize the genes that represent their epigenetic functions. Expression levels were quantified using *M. fascicularis* gene annotations. A quantitative matrix was constructed using TopHat2 (TopHat v2.0.13) and Cuffdiff2 (v2.2.1), and differentially expressed genes (DEGs) were examined (Trapnell et al., 2012). A total of 22,529 genes were selected from paralogues using the human gene annotation database; of those, 10,458 genes were remained to be functionally important in primates based on LoFtool score, which is based on loss-of-function variants, synonymous mutations, *de novo* mutation rate, and evolutionary conservation (Fadista et al., 2016). LoFtool score was predicted to search for genetic diseases in particularly detecting neurodevelopmental disorder and investigating complex brain diseases with strong genetic effects. To investigate genes specific to each brain region, DEGs were identified between conditions. These DEGs were examined using Cuffdiff default options, and the significantly changed genes in each brain region were represented as a network (Fisher’s exact test; *q*-value ≤ 0.05). The R package ComplexHeatmap (v1.17.1) was used to identify differences in gene expression patterns (Gu et al., 2016). Each region was normalized against the trimmed mean of *M*-values (TMM) algorithm (Robinson and Oshlack, 2010). To filter the characteristics of clusters, a fold change of 4 was selected from the available options (2, 4, 8, 16, and 64) (Lin et al., 2014). The normalized FPKMs were converted into standard *z*-scores; if a value was greater than 2 or less than −2, it was replaced with 2 or −2, respectively. A heatmap was generated by Euclidean clustering with default options. To analyze functional annotation in each of the clustered genes, we performed gene set enrichment analysis (GSEA) by using PathwayStudio (Elsevier, Amsterdam, the Netherlands).

### Correlating Gene Expression With DNA Methylation Level

To identify genes affected by DNA methylation in the crab-eating monkey, Pearson’s correlation was calculated between DNA methylation and gene expression levels. CpG islands (CGIs) were defined in promoter regions, and the methylation levels of the corresponding CGIs were calculated using the median DNA methylation level for all sites in the intersecting region. The matrix was statistically tested using in-house scripts, revealing 92 significantly correlated genes (Pearson’s correlation test; *p*-value ≤ 0.05). These genes were used to conduct a GSEA using Ingenuity Pathway Analysis (IPA; QIAGEN Inc., https://www.qiagenbioinformatics.com/products/ingenuitypathway-analysis) (Krämer et al., 2013). Also, the significant genes were divided into two groups (positively and negatively correlated), and canonical pathways were investigated.

### Repeat Annotation and Identification of DNA Methylation Patterns

RepeatMasker results in the table browser of UCSC Genome Browser were used to classify repeat elements according to repeat types and subtypes. To calculate DNA methylation according to the genomic features of repeat elements, genomic features were identified using BEDTools. Using the R package EnrichedHeatmap (v1.9.4), repeats were separated into subtypes and smoothed using the LOESS algorithm to find patterns (Gu et al., 2018). In-house scripts were used to compare the internal and external regions of the repeats.

## Results

### Processing of RNA-seq and WGBS Data From Seven Brain Regions

For integrated analysis of the DNA methylome and transcriptome of the crab-eating monkey, we generated RNA-seq and WGBS datasets from seven different brain regions (angular gyrus, anterior caudate, cingulate gyrus, hippocampus middle, inferior temporal lobe, dorsolateral prefrontal cortex, and ventrolateral prefrontal cortex). To confirm correlations between samples, we performed Pearson’s correlation analysis of gene expression values (**Additional file 1: Table S4**; *p*-value < 2.2e-16). Correlations were greater than 0.95 for every paired set, except for anterior caudate and ventrolateral prefrontal cortex (**Additional file 1: Table S4**). We used 35,141 CpG sites to calculate Pearson’s correlation values from the quantified DNA methylation levels of brain regions (**Additional file 1: Table S5**; *p*-value < 2.2e-16). Every paired set had *r*-values greater than 0.98, and the highest *r* value was observed for inferior temporal lobe and dorsolateral prefrontal cortex.

### Functional Characteristics Distinguished by Brain Region-Specific Gene Expression Patterns in *M. fascicularis*

**Figure 1:**
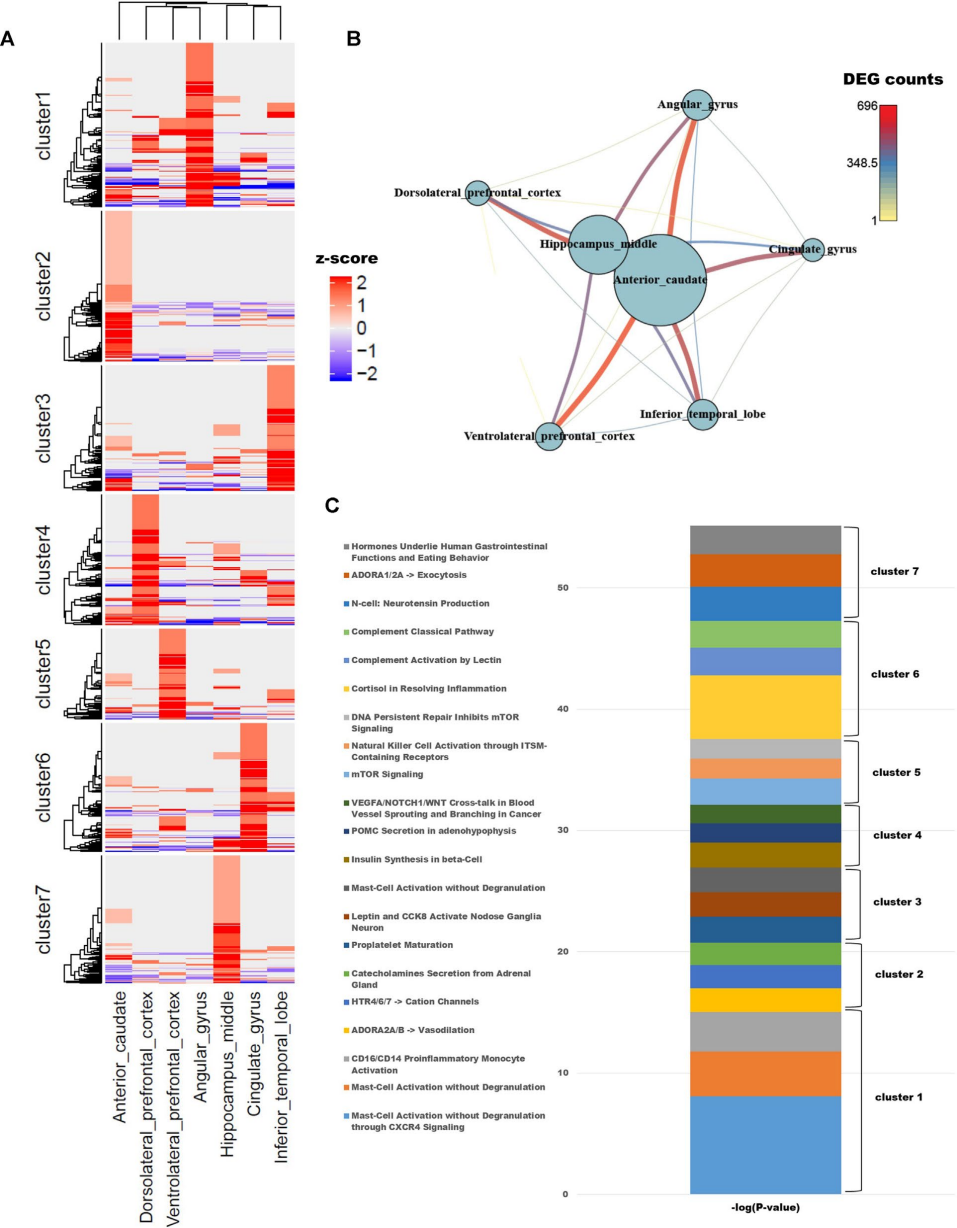
**(A)** Unsupervised *k*-means clustering of brain regions from crab-eating monkey. The *z*-scores were converted from the log_2_(FPKM + 1) values and were limited to the range [−2, 2]. **(B)** The network represents the number of differentially expressed genes (DEGs). Nodes represent the total number of DEGs in each of the seven brain regions, and edges represent the number of DEGs in the corresponding connection. **(C)** Biological process were investigated from unsupervised clustered gene sets. The *p*-values in the pathway terms were converted to –log (*p*-values), and the significance of each cluster is expressed in a cumulative graph.

To identify differences in gene expression among brain regions, we examined genes with known functions in humans. To functionally characterize monkey brain regions in terms of the corresponding human brain regions, a total of 10,458 genes with known functions were selected based on their LOFtool score (Fadista et al., 2016). Pairwise comparison of brain identified 1,418 genes with a fold change ≥ 4 in at least one pair. We performed *k*-means clustering using the PAM algorithm to identify genes that were highly expressed in each region (**Additional file 1: Table S1**; *k* = 7). Each cluster contained approximately 200 genes, with the exception of cluster 5, which contained only 139. Each region had one cluster with a group of particularly high values (**Figure 1A**). GSEA was carried out for each cluster group, which represented genes expressed in each brain region. In addition, we used PathwayStudio to investigate the biological processes of the pathways involving the clustered genes (**Figure 1C**).

Cluster 1, a gene set specifically expressed in angular gyrus, is activated in mast cells during inflammation and also plays an important role in migraine, a primary headache disorder (Boes and Levy, 2012; Pavlovic et al., 2017). Cluster 2, associated with anterior caudate, is enriched in genes involved in vasodilation and catecholamine secretion from the adrenal gland cluster 3, expressed in the inferior temporal lobe, and contained three genes (*PCSK1*, *CARTPT*, and *JAK2*) associated with leptin and CCK8 activation in inferior (nodose) ganglia neuron; the members of this cluster are related to genes expressed in neuroendocrine cells, neuropeptides, and diseases of clonal eosinophilia (Seidah et al., 1991; Douglass and Daoud, 1996; Lacronique et al., 1997; Reiter et al., 2005; Reiter and Gotlib, 2017). Cluster 4, expressed at high levels in dorsolateral prefrontal cortex, is enriched in genes involved in insulin synthesis; obesity and insulin resistance are closely related to the functions of the hippocampus, angular gyrus, and dorsolateral prefrontal cortex (Cheke et al., 2017). Cluster 5, which as noted above contained the smallest number of genes, is associated with mTOR signaling and NK cell activation, both of which are related to the brain immune system. Cluster 6, expressed in cingulate gyrus, is enriched in terms similar to those associated with cluster 5: the immune system, classical complement pathway, complement activation by lectin, and cortisol in inflammation. Cluster 7, expressed in hippocampus middle, is enriched in genes related to neurotensin production and exocytosis; in particular, neuromedin N and neurotensin, which are associated with gastrointestinal function and eating behavior, were highly expressed.

To compare the number of DEGs among the seven brain regions, we depicted them as a network (**Figure 1B**); this analysis revealed that hippocampus middle and anterior caudate had larger numbers of DEGs than the other regions. On the other hand, dorsolateral prefrontal cortex had fewer DEGs than all other regions except for hippocampus middle and anterior caudate, and only a few significant DEGs relative to ventrolateral prefrontal cortex.

### Annotation of DNA Methylation Variable Sites and Genomic Functions

**Figure 2:**
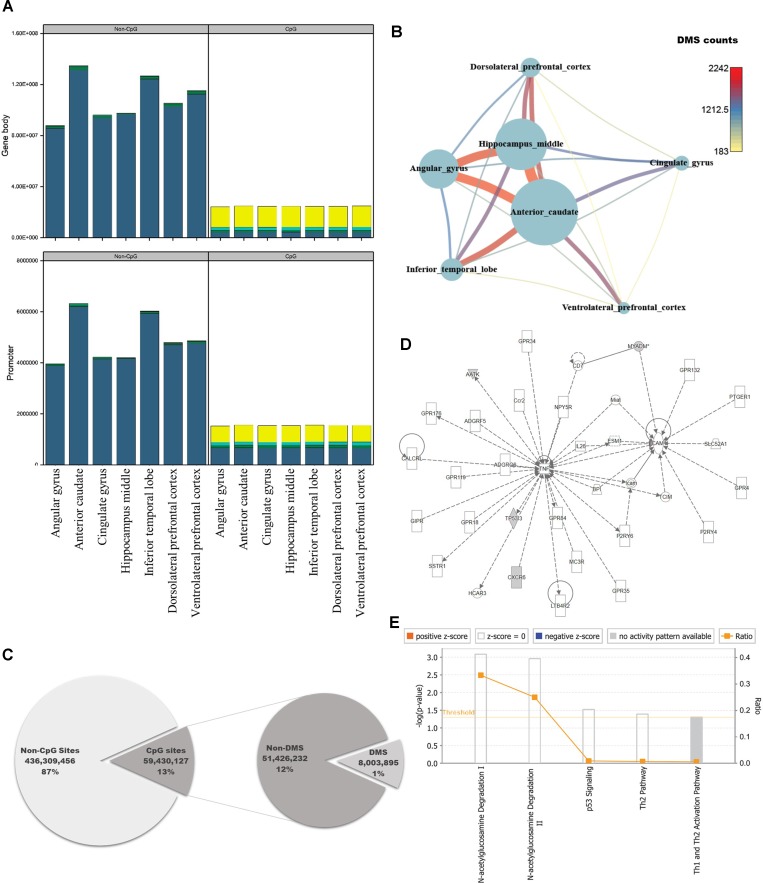
Quantification of DNA methylation in each of the seven brain regions, and comparison of differentially methylated sites among regions. **(A)** Proportion of DNA methylation levels in each sample by different genomic features (DNA methylation level: bright yellow: 0–25%; cyan: 25–50%; green: 50–75%; dark blue: 75–100%). **(B)** Network created using the numbers of differentially methylated sites (DMSs) in seven brain regions. Nodes represent the total numbers of DMSs identified by pairwise comparisons of the seven brain regions, and edges are the numbers of DMSs relative to other brain regions. **(C)** Pie chart representing regions in the genome classified according to genomic features, with statistical significance. **(D)** Causal network from six significantly differentially methylated genes. **(E)** Canonical pathways for six significantly differentially methylated genes.

The brain is the most complex organ in animals, but DNA methylation in the brain has not been fully characterized at the genomic level. Previous studies identified approximately 1,500 CpG sites in human brain, using array-based approaches or analyzing limited regions of the genome (Ladd-Acosta et al., 2007; Zhang et al., 2010; Ladd-Acosta et al., 2014). However, because we could use information from the whole genome, we performed pairwise comparisons of DNA methylation patterns in brain regions from crab-eating monkey, with the goal of identifying differentially methylated genes (DMGs) associated with brain function. Genes were defined as DMGs if their first exons overlapped with CGIs containing differentially methylated sites (DMSs). The average DMSs in the brain regions among the total CpG sites were 5,277, and the average in promoter regions is 417 (**Additional file 1: Table S7**). In particular, genes with significantly altered DNA methylation levels were classified as DMGs if the first exon, which has an epigenetic effect on gene structure, overlapped with CGIs under any condition. DNA methylation status of cytosines was calculated at an average of 258 million sites per brain region using WGBS data. We investigated the number of computable DNA methylation sites of each brain regions, and the sites were categorized by genomic features as promoter and gene body. In addition, the featured sites were grouped by CpG or non-CpG sites. Promoter and gene body regions contained a similar proportion of DNA methylation levels (**Additional file 1: Table S6**, **Figure 2A**). The proportion of hypo-methylated DNA sites in non-CpG sites was very low, and CpG sites have shown that the hypo-methylated DNA sites in gene body are higher than promoter. Eight million sites had significant differences from 59 million CpG sites of the total 495 million cytosines (**Figure 2C**; Fisher’s exact test; *p*-value ≤ 0.05). Comparison of the DNA methylated sites among the seven brain regions yielded an average of 738 DMSs in each pair of regions (Fisher’s exact test; *q*-value ≤ 0.05). The number of DMSs was expressed as a network (**Figure 2B**), which exhibited patterns very similar to those in the DEG network (**Figure 1B**). The smallest number of DMSs (183 sites) were detected between dorsolateral prefrontal cortex and ventrolateral prefrontal cortex. The regions with the largest difference in DMSs were the same as the regions with the largest difference in DEGs, i.e., the anterior caudate and hippocampus middle (2,242 sites).

To investigate the genomic functions of DMSs, we selected significant genes, ultimately identifying 11 DMGs in brain regions (Fisher’s exact test; *q*-value ≤ 0.01). We investigated enriched terms associated with DMGs, along with six gene symbols that shared names with human genes. We found that they were involved in brain functions and epigenetic regulation, as determined by Gene Ontology analysis using DAVID (**Figures 2D, E**; **Table S8**) (Dennis et al., 2003). CXCR6, which encodes the C-X-C chemokine receptor type 6 protein, is the coreceptor used when HIV-1 and SIV enter target cells and is associated with HIV-associated dementia (Cilliers et al., 2003; Cartier et al., 2005). AMDHD2 is a protein with hydrolase activity and N-acetylglucosamine-6-phosphate deacetylase activity. AATK is a serine/threonine also known as apoptosis-associated tyrosine kinase. Several studies have examined epigenetic reprocessing through microRNA; among neurodegenerative disorders, Alzheimer’s disease, schizophrenia, and bipolar disorder have been examined in this manner (Shioya et al., 2010; Moreau et al., 2011; O’Carroll and Schaefer, 2013). MYADM, the myeloid-associated differentiation marker, is highly expressed in heart, brain, placenta, lung, and pancreas (O’Carroll and Schaefer, 2013). Hyper-methylated CpG islands were observed in the promoter region in hepatocellular carcinoma (Song et al., 2013). TP53I3, which encodes a putative quinone oxidoreductase, mediates p53-mediated cell death; it is differentially expressed in ganglioglioma and is also associated with several types of brain tumors (Fassunke et al., 2008; Alajez et al., 2009; Ghassemifar and Mendrysa, 2012; Schulten et al., 2017). IPA revealed that four of the six genes (AATK, CXCR6, MYADM, and TP53I3) were enriched in a single network involved in N-acetylglucosamine degradation (**Figures 2D, E**). Notably in this regard, O-linked β-N-glucosamine is a post-translational protein modification that plays important roles in brain functions (Iyer and Hart, 2003; Lamarre-Vincent and Hsieh-Wilson, 2003).

### Genes Correlated With DNA Methylation Levels are Associated With the Function and Development of the Brain

**Figure 3:**
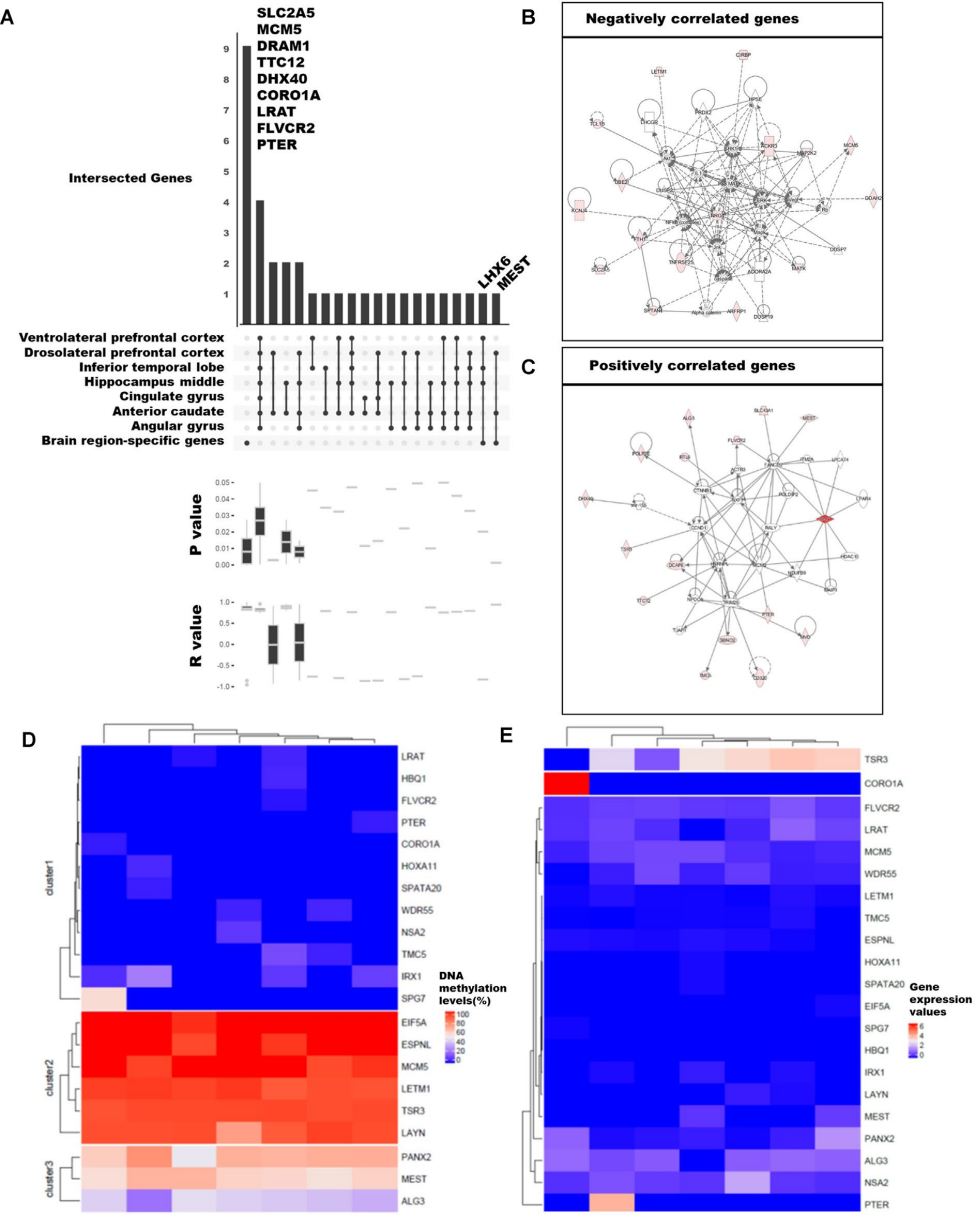
Categorization of genes with strong correlation between DNA methylation and expression levels. **(A)** Classification of 89 genes with brain region–specific characteristics and differentially methylated genes (DMGs). Sites with significant changes (Fisher’s exact test; *q*-value ≤ 0.05) in CpG islands (CGIs) within the 2-kb sequence upstream of the gene were defined as DMGs, and the brain regions were pointed in a dot below. The *p*-values and *r*-values of the genes in the corresponding categories were determined by boxplot using Pearson’s correlation test. **(B)**, **(C)** Gene set enrichment network using Ingenuity Pathway Analysis (IPA) of positively and negatively correlated genes. We revealed the correlation from 92 genes and finally selected 21 genes (Pearson’s correlation test; *p*-value ≤ 0.01). The DNA methylation level **(D)** and gene expression values **(E)** were shown in heatmaps. The columns ordered in the heatmaps from left to right were ventrolateral prefrontal cortex, hippocampus middle, cingulate gyrus angular gyrus, anterior caudate, dorsolateral prefrontal cortex, and inferior temporal lobe.

We examined the differences in gene expression and DNA methylation levels and searched for the relationship of genes that were related. The association between gene expression and DNA methylation was examined using 75,045 CpG islands and 62,757 transcripts. A total of 59 million DNA methylation sites were used to obtain the median values for CpG islands. We selected 3,261 CpG islands that overlapped with the promoter regions of the 20,579 genes with known functions. We then calculated Pearson’s correlations between gene expression values and DNA methylation levels. Ultimately, 92 significant pairs of CpG islands and gene expression patterns were identified (**Figures 3A–C**; *p*-value ≤ 0.05), of which 53 were positively correlated and 39 were negatively correlated; remarkably, the number of positively correlated pairs was larger.

Among the 92 genes with strong correlations, we examined DMGs with DMSs in the CpG islands overlapping the first exon of highly expressed brain region-specific genes (BRSGs) (**Figure 3A**). Nine genes (*SLC2A5*, *MCM5*, *DRAM1*, *TTC12*, *DHX40*, *COR01A*, *LRAT*, *FLVCR2*, and *PTER*) exhibited correlations with BRSG characteristics. *SLC2A5* is specifically expressed in the blood–brain barrier (Dahlin et al., 2009). *MCM5* is a proliferation marker in brain tumors (Ohta et al., 2001), and expression of *DRAM1* is related to neuronal autophagy (Crighton et al., 2006; Galavotti et al., 2013). These genes were hyper-methylated in all brain regions (**Figure 3D**), and their expression levels were also low (**Figure 3E**). *TTC12* is a brain biomarker in alcoholism (Yang et al., 2008), and numerous studies have been performed on polymorphisms in this gene (Gelernter et al., 2006; Nelson et al., 2013). *DHX40* is a DEG in cerebellum of *Neurog1* null mice (Dalgard et al., 2011). *LRAT* encodes an endoplasmic reticulum protein that plays an important role in the visual system; mutation in this gene is associated with severe early-onset retinal dystrophy. This gene was hypo-methylated in all brain regions, and its expression levels were lower than those of other correlated genes (**Figure 3E**). *FLVCR2* is expressed in the central nervous system, vessels of the matured retina, and fetal pituitary during embryonic development (Brasier et al., 2004). This gene was methylated in nearly all brain regions (**Figure 3D**), and its expression levels were low relative to other genes. *PTER* encodes a phosphotriesterase-related protein associated with Waardenburg syndrome type 2c (WS2C); affected tissues include skin and eye (Selicorni et al., 2002; Pingault et al., 2010). This gene was hypo-methylated in all brain regions (**Figure 3D**) and was highly expressed only in inferior temporal lobe (**Figure 3E**).

Mutation and gene expression studies have shown that the nine genes correlated with BRSG characteristics have effects in brain or eye tissue. In particular, two of these genes, *LHX6* and *MEST*, were both BRSGs and DMGs. *LHX6*, an epigenetic marker of diverse neurons in mammalian brain (Mo et al., 2015), as well as head and neck carcinomas (Estécio et al., 2006), encodes a protein that functions as a transcriptional regulator in differentiation and development of neural and lymphoid cells. *MEST* is a maternally imprinted gene located in a human autism susceptibility locus; changes in DNA methylation in its promoter affect its expression in the cortex of human, chimpanzee, old world monkey (baboon, rhesus macaque), and new world monkey (marmoset) (Schneider et al., 2012). A moderate level of DNA methylation (**Figure 3D**) and relatively low levels of gene expression were observed (**Figure 3E**).

Among the genes with correlations to BRSG characteristics, we identified eight positively correlated genes (*DRAM1*, *TTC12*, *DHX40*, *CORO1A*, *MEST*, *LRAT*, *FLVCR2*, and *PTER*) and three negatively correlated genes (*SLC2A5*, *MCM5*, and *LHX6*) (**Figures 3B, C**). Of the positively correlated genes, *TTC12*, *DHX40*, *MEST*, and *FLVCR2* are involved in embryonic development, organ development, and organismal development in the same network (**Figure 3C**). In addition, *SPG7*, which was highly methylated in ventrolateral prefrontal cortex, is an alternatively methylated gene involved in neurological disease (**Figures 3C, D**). Among the negatively correlated genes, we identified a network containing *SLC2A5* and *MCM5* (**Figure 3B**) that was associated with the cell cycle, cell–cell signaling and interaction, and drug metabolism.

### Characteristics of DNA Methylation According to Annotation of Repetitive Elements Repeat Type

**Figure 4:**
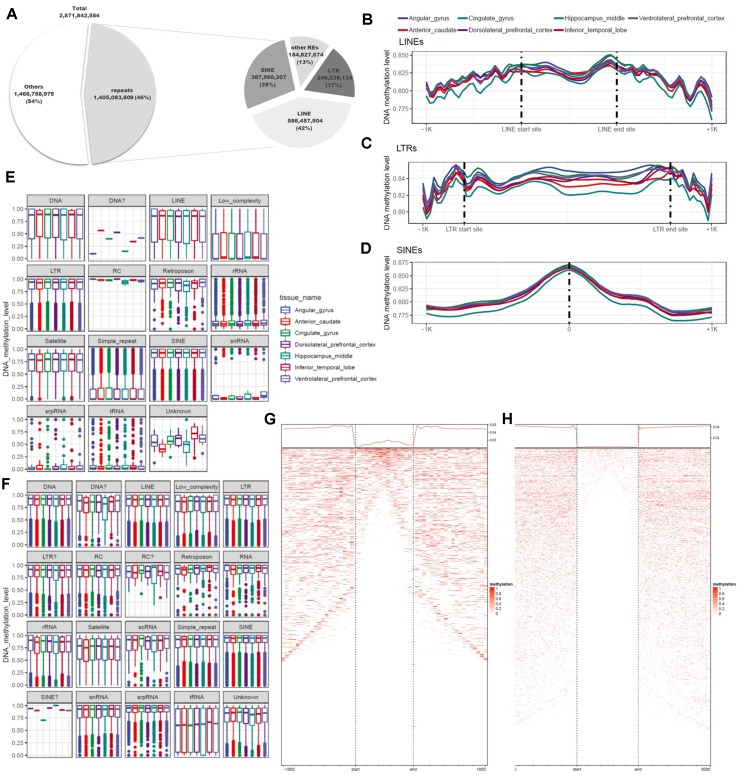
Differences in genomic features and DNA methylation level according to type of repeat element. **(A)** Proportions of repeats, according to genomic features and repeat type. **(B)** Trend in DNA methylation level in the inner and outer regions of long interspersed nuclear elements (LINEs). **(C)** Trend in DNA methylation level in the inner and outer regions of long terminal repeats (LTRs). **(D)** Distribution of DNA methylation in the center of short interspersed nuclear elements (SINEs). **(E)** Distribution of repeat elements, including CpG islands. **(F)** Distribution of repeat elements, excluding CpG islands. **(G)** Trends and row-sorted heatmap of LTRs. **(H)** Trends and row-sorted heatmap of LINEs.

DNA methylation varies among REs and plays diverse roles throughout the genome. Previous studies used methods based on antibodies or restriction sites to analyze DNA methylation, but the whole genome was not fully covered by these approaches. In this study, we used WGBS, which can detect methylation at the whole-genome level, making it possible to examine DNA methylation at single-base resolution in non-CpG regions of REs. To identify the functions of REs in epigenetic regulation, we conducted a quantitative analysis of DNA methylation based on annotation of REs. For this purpose, 5,174,162 REs were created by RepeatMasker used in the UCSC Genome Browser. A total of 1.4 billion base pairs were annotated, of which 87% were LTRs, LINEs, or SINEs (**Figure 4A**; **Additional file 1: Table S9**). We separated these sequences into two groups based on the presence or absence of CGIs, and then characterized the distributions of DNA methylation (**Figures 4E, F**). CpG and non-CpG groups both tended to be hyper-methylated in LTRs, LINEs, and SINEs. The median DNA methylation value in CpG groups tended to be lower in LINEs than in other repeat elements (**Figure 4A**). Simple repeats were hypo-methylated in the CpG group, but hyper-methylated in the non-CpG group. We also observed a similar pattern in low-complexity repeats. Satellites exhibited a pattern similar to those of other hyper-methylated repeat elements, but the median value was relatively low. LINEs, which constituted the largest percentage of repeat elements, had higher DNA methylation levels at both ends of the repeat region (**Figures 4B, F**), consistent with previously reported findings (Papin et al., 2017). The average difference in DNA methylation level in the inner region of the repeat element was 0.826 (standard deviation, 0.247), a difference of 0.024 relative to the average of 0.850 in the outer 100-bp region. In the LTR, the mean and standard deviation of the repeat region were 0.846 and 0.221, respectively, a difference of 0.010 relative to the averages of 0.856 and 0.215 on the outside (**Figures 4B, C**). In SINEs, the DNA methylation level increased in the center of the repeat region: 0.875 ± 0.204 in the interior *vs*. 0.815 ± 0.262 in the outer region (**Figure 4D**). We observed that the hyper-methylated region was noticeably smaller inside the repeat region (**Figures 4G, H**). SINEs are the most well conserved repeat elements among REs, which contain pan-conserved sequences (Papin et al., 2017) and are hyper-methylated (Varshney et al., 2015). Because the types of repeats are distinguished by the size, DNA methylation differs for each subtype, and variation exists in both size and number (**Additional file 1: Figure S1b**). However, in the case of MIRc, a pan-mammalian SINE, DNA was hypo-methylated (**Additional file 1: Figure S1a**). AluJr4 was hypo-methylated, unlike other Alus, and AluSx4 and AluJo had relatively low DNA methylation values but were not considered hypo-methylated.

## Discussion

The role of epigenetic regulation of DNA methylation has been studied under a variety of conditions in many species (Gao and Das, 2014). In the human brain, the dynamics of DNA methylation are closely related to learning and memory-related processes, experience-dependent plasticity in the visual cortex, and age (Tognini et al., 2015; Yang et al., 2015). However, previous studies obtained results that were restricted by methodological limitations or sample characteristics (Gu et al., 2011; Taiwo et al., 2012). In particular, RRBS and MeDIP-seq, which only examine limited regions, have been widely used in DNA methylation studies (Davies et al., 2012; Illingworth et al., 2015; Zhang et al., 2016). Consequently, we know very little about non-CpG sites and sites that cannot be captured by methyl-binding proteins. On the other hand, WGBS has the ability to investigate DNA methylation in understudied regions throughout the genome (Yong et al., 2016). However, most previous WGBS studies were conducted using samples from distinct developmental stages or diseased tissues, and very few analyzed normal adult brain tissue (Heyn et al., 2012; Habibi et al., 2013; Smallwood et al., 2014).

The crab-eating monkey is widely used in pharmacological experiments, but its epigenetic characteristics have rarely been studied. In this study, we created an epigenetic map of a crab-eating monkey, and then elucidated its characteristics at the whole-genome level. However, this study had several limitations. First, the brain regions that we examined were acquired from a single individual, and we examined epigenetic changes and functions only in these regions. Consequently, our data cannot address inter-individual variation. Also, because we used bulk tissues, we were unable to detect cell type-specific features. Nevertheless, by investigating DNA methylation and gene expression patterns of specific brain regions, we identified 92 genes with strong correlations. Based on mutation studies or brain region-specific patterns of expression, nine of these genes are known to affect regulation of the brain or eye, or to be involved in diseases and development of these tissues. Additionally, among the brain region-specific genes, we identified two promoter regions (*LHX6* and *MEST*) that exhibited significant changes in DNA methylation level among brain regions, and therefore represent brain-specific epigenetic markers. In future studies, these results could be combined with data from with other individuals, different genders, or other developmental stages.

Comparisons of differences in gene expression and DNA methylation revealed a strong correlation (≥0.90), which we interpreted as an indication that the differences between samples were modest (**Additional file 1: Table S5**). Although the differences at the genome-wide level were not significant, we did identify differences at the gene level. Remarkably, this revealed a single enriched pathway related to brain function. Thus, we found genes related to region-specific developmental pathways or functions (**Figures 2D, E**; **Additional file 1: Table S8**). We analyzed these patterns to find links between gene expression and DNA methylation. The relationship was examined using other, non-brain tissues, and we observed a distinct negative correlation that was not statistically significant. In addition, we observed a weak positive correlation among all samples. Genes with significant statistical differences were also associated with brain functions and development, or with epigenetic regulation. Other studies using brain tissues revealed many genes with positive correlations (Gibbs et al., 2010; Davies et al., 2012; Chen et al., 2014; Zhang et al., 2016).

DNA methylation has also been extensively studied in repeat elements, and its functions vary according to the type and subtype of repeats (Bogdanović et al., 2011; Guo et al., 2014; Wang et al., 2014). Criteria for dividing repeat types and subtypes focused on size, so that we could identify characteristics that varied among subtypes within a given repeat type (Vassetzky and Kramerov, 2012). Our results revealed that epigenetic status differs according to repeat subtype, consistent with findings obtained previously using the read-count method. These observations showed that patterns associated with the genomic feature of repeats were constant. However, the average DNA methylation values in all regions were ≥0.75, and the average differences were not remarkable. This may have been due to the reduction in the number of hyper-methylated sites within the repeat inner region (**Figures 4G, H**). In addition, we observed non-hyper-methylated repeats, suggesting that transcripts in these repeat elements might be expressed.

In conclusion, our analysis of the adult brain regions of a crab-eating monkey by RNA-seq and WGBS reveal the roles of DNA methylation from multiple perspectives. Our findings should facilitate future studies of the relationship between epigenetics and brain function.

## Data Availability

The datasets supporting the results of this article are available in the European Nucleotide Archive (ENA). The WGBS and RNA-seq libraries for all seven brain regions are deposited under accession number PRJEB31707.

## Ethics Statement

Ethical approval for collecting blood samples from cynomolgus macaque was granted by the Institutional Animal Care and Use Committee (IACUC) and the Korea Institute of Toxicology (Approval No: 110-0223). Animal preparation and experiments were conducted in accordance with protocols approved by the guidance of the IACUC.

## Author Contributions

NK and YK designed and supervised this project. W-JL, and KK analyzed the data and drafted the manuscript. J-YK interpreted and visualized the results. SY, J-HO, and J-WC prepared the brain region samples. H-JK, MK, and J-LP performed the WGBS and RNA-seq library construction. All authors reviewed and approved the final manuscript.

## Funding

This work was supported by grants from the National Research Foundation of Korea (NRF-2014M3C9A3064552) and the KRIBB Initiative program.

## Conflict of Interest Statement

The authors declare that the research was conducted in the absence of any commercial or financial relationships that could be construed as a potential conflict of interest.

## Abbreviations

WGBS, whole-genome bisulfite sequencing; CGI, CpG island; FPKM, fragments per kilobase of exon per million mapped fragments; GSEA, gene set enrichment analysis; DEG, differentially expressed gene; BRSG, brain region-specific gene; DMS, differentially methylated site; DMG, differentially methylated gene; TSS, transcription start site; TES, transcription end site; RE, repeat element; LTR, long terminal repeat; LINE, long interspersed nuclear element; SINE, short interspersed nuclear element.

## Supplementary Material

Table S1 Reference alignment statistics of whole-genome bisulfite sequencing.

Table S2 Methylation calling statistics.

Table S3 Reference alignment statistics of whole-transcriptome sequencing.

Table S4 Pearson’s correlation r values of gene expression matrices from each sample.

Table S5 Pearson’s correlation r values of DNA methylation matrices from each sample.

Table S6 Coverage of CpG sites of sequenced reads.

Table S7 Simple statistics of DMS counts by different genome features.

Table S8 DAVID annotation and GO terms from 11 DMGs.

Table S9 Summary statistics of repeat elements.

Figure S1 DNA methylation status and size of SINEs. a. Box plot of DNA methylation of SINEs, by repeat type. b. Box plot of size of each type of SINE.

Table 2 Additional file 2; The results of brain region-specific genes.

Table 3 Additional file 3; The results of Pearson's correlation between gene expression and DNA methylation levels.
