# Supplementary material for: Investigation of Gene Expression and DNA Methylation From Seven Different Brain Regions of a Crab-Eating Monkey as Determined by RNA-Seq and Whole-Genome Bisulfite Sequencing
Source: Front Genet. 2019 Jul 26;10:694. doi: 10.3389/fgene.2019.00694 (PMC6690020; doi:10.3389/fgene.2019.00694)
Supplement: Table S1 — Reference alignment statistics of whole-genome bisulfite sequencing. [file Table_1.docx]

|  | |  |  |  |  |
| --- | --- | --- | --- | --- | --- |
|  | **Reference alignments** | | | | |
| **Sample name** | **total_reads** | **aligned_reads** | **no_alignments** | **ambig_reads** | **percent_aligned** |
| **Angular gyrus** | 350,230,270 | 256,256,588 | 81,572,627 | 12,401,055 | 72.96% |
| **Anterior caudate** | 442,688,233 | 324,411,198 | 102,546,008 | 168,568,102 | 73.43% |
| **Cingulate gyrus** | 344,531,942 | 249,361,141 | 82,273,368 | 12,897,433 | 72.26% |
| **Hippocampus middle** | 383,521,964 | 274,357,687 | 94,414,836 | 151,561,390 | 71.46% |
| **Inferior temporal lobe** | 433,094,362 | 318,155,531 | 99,827,649 | 15,111,182 | 73.48% |
| **Dorsolateral prefrontal cortex** | 377,724,526 | 274,180,429 | 90,373,594 | 158,104,767 | 72.28% |
| **Ventrolateral prefrontal cortex** | 349,876,223 | 253,724,028 | 85,374,790 | 10,777,405 | 72.79% |

Table S1. Reference alignment statistics of whole-genome bisulfite sequencing

|  | **Methylation calling** | | | | | | |
| --- | --- | --- | --- | --- | --- | --- | --- |
| **Sample name** | **total_c** | **meth_chg** | **meth_chh** | **meth_cpg** | **unmeth_chg** | **unmeth_chh** | **unmeth_cpg** |
| **Angular gyrus** | 9,590,683,965 | 80,746,747 | 219,889,131 | 436,696,255 | 2,229,413,861 | 6,465,324,036 | 158,613,935 |
| **Anterior caudate** | 13,072,567,117 | 105,445,864 | 278,220,021 | 597,853,376 | 3,053,824,407 | 8,773,849,690 | 263,373,759 |
| **Cingulate gyrus** | 9,553,623,866 | 76,718,670 | 208,347,007 | 433,026,961 | 2,219,133,128 | 6,449,062,002 | 167,336,098 |
| **Hippocampus middle** | 10,493,855,909 | 60,660,548 | 145,410,168 | 469,874,340 | 2,458,904,502 | 7,164,987,238 | 194,019,113 |
| **Inferior temporal lobe** | 12,692,698,946 | 95,343,704 | 242,808,655 | 592,238,998 | 2,991,493,555 | 8,531,520,969 | 239,293,065 |
| **Dorsolateral prefrontal cortex** | 10,400,172,463 | 84,449,201 | 230,217,767 | 473,539,589 | 2,426,878,158 | 7,003,005,526 | 182,082,222 |
| **Ventrolateral prefrontal cortex** | 10,295,263,918 | 89,528,730 | 260,349,930 | 452,039,009 | 2,348,461,919 | 6,985,954,265 | 158,930,065 |

Table S2. Methylation calling statistics

| **Sample name** | **forward reads** | **reverse reads** | **total reads** | **mapped reads** | **exon contents** |
| --- | --- | --- | --- | --- | --- |
| **Angular_gyrus** | 72,160,955 | 72,162,910 | 144,323,865 | 103,705,504 | 71.86% |
| **Anterior_caudate** | 94,884,730 | 94,886,312 | 189,771,042 | 114,999,490 | 60.60% |
| **Cingulate_gyrus** | 75,004,070 | 75,005,239 | 150,009,309 | 101,585,846 | 67.72% |
| **Hippocampus_middle** | 65,445,416 | 65,446,508 | 130,891,924 | 91,110,781 | 69.61% |
| **Inferior_temporal_lobe** | 55,058,014 | 55,058,991 | 110,117,005 | 75,842,740 | 68.87% |
| **Dorsolateral_prefrontal_cortex** | 59,653,104 | 59,655,251 | 119,308,355 | 80,551,478 | 67.52% |
| **Ventrolateral_prefrontal_cortex** | 85,042,290 | 85,044,982 | 170,087,272 | 120,415,098 | 70.80% |

Table S3. Reference alignment statistics of whole-transcriptome sequencing

| **Sample name** | **Angular gyrus** | **Anterior caudate** | **Cingulate gyrus** | | | **Hippocampus middle** | **Inferior temporal lobe** | **Dorsolateral prefrontal cortex** | **Ventrolateral prefrontal cortex** |
| --- | --- | --- | --- | --- | --- | --- | --- | --- | --- |
| **Angular gyrus** | **1.000** | **0.952** | | **0.981** | **0.965** | | **0.976** | **0.984** | **0.983** |
| **Anterior caudate** | **0.952** | **1.000** | | **0.953** | **0.950** | | **0.953** | **0.954** | **0.947** |
| **Cingulate gyrus** | **0.981** | **0.953** | | **1.000** | **0.969** | | **0.982** | **0.983** | **0.981** |
| **Hippocampus middle** | **0.965** | **0.950** | | **0.969** | **1.000** | | **0.969** | **0.968** | **0.964** |
| **Inferior temporal lobe** | **0.976** | **0.953** | | **0.982** | **0.969** | | **1.000** | **0.979** | **0.977** |
| **Dorsolateral prefrontal cortex** | **0.984** | **0.954** | | **0.983** | **0.968** | | **0.979** | **1.000** | **0.983** |
| **Ventrolateral prefrontal cortex** | **0.983** | **0.947** | | **0.981** | **0.964** | | **0.977** | **0.983** | **1.000** |

Table S4. Pearson’s correlation r values of gene expression matrices from each sample

| **Sample name** | **Angular gyrus** | **Anterior caudate** | **Cingulate gyrus** | **Hippocampus middle** | **Inferior temporal lobe** | **Dorsolateral prefrontal cortex** | **Ventrolateral prefrontal cortex** |
| --- | --- | --- | --- | --- | --- | --- | --- |
| **Angular gyrus** | **1.000** | **0.985** | **0.986** | **0.982** | **0.989** | **0.988** | **0.987** |
| **Anterior caudate** | **0.985** | **1.000** | **0.988** | **0.985** | **0.990** | **0.989** | **0.987** |
| **Cingulate gyrus** | **0.986** | **0.988** | **1.000** | **0.986** | **0.990** | **0.989** | **0.988** |
| **Hippocampus middle** | **0.982** | **0.985** | **0.986** | **1.000** | **0.988** | **0.986** | **0.985** |
| **Inferior temporal lobe** | **0.989** | **0.990** | **0.990** | **0.988** | **1.000** | **0.991** | **0.990** |
| **Dorsolateral prefrontal cortex** | **0.988** | **0.989** | **0.989** | **0.986** | **0.991** | **1.000** | **0.989** |
| **Ventrolateral prefrontal cortex** | **0.987** | **0.987** | **0.988** | **0.985** | **0.990** | **0.989** | **1.000** |

Table S5. Pearson’s correlation r values of DNA methylation matrices from each sample

| **sample names** | **average depth** | **total CpG sites** | **remained CpG sites*** | **remained rates(%)** |
| --- | --- | --- | --- | --- |
| **Angular gyrus** | 7.24664 | 49,340,054 | 13,481,617 | 27.32% |
| **Anterior caudate** | 9.64544 | 50,627,365 | 20,313,194 | 40.12% |
| **Cingulate gyrus** | 7.51651 | 50,234,355 | 14,592,056 | 29.05% |
| **Hippocampus middle** | 7.5906 | 50,351,820 | 14,761,085 | 29.32% |
| **Inferior temporal lobe** | 9.331 | 50,090,328 | 19,358,001 | 38.65% |
| **Dorsolateral prefrontal cortex** | 8.0088 | 50,241,112 | 16,064,476 | 31.97% |
| **Ventrolateral prefrontal cortex** | 8.03718 | 51,159,246 | 16,598,096 | 32.44% |

*sites with read depth ≥ 10.

Table S6. Coverage of CpG sites of sequenced reads

| **Sample names** | **CpG total** | **CpG promoter** | **CpG exon** | **CpG intro** | **CpG intergenic** |
| --- | --- | --- | --- | --- | --- |
| **Angular gyrus** | 6,454 | 463 | 2,695 | 1,945 | 1,814 |
| **Anterior caudate** | 11,015 | 889 | 4,312 | 3,129 | 3,574 |
| **Cingulate gyrus** | 2,215 | 171 | 901 | 665 | 649 |
| **Hippocampus middle** | 8,499 | 602 | 3,454 | 2,405 | 2,640 |
| **Inferior temporal lobe** | 3,725 | 369 | 1,403 | 956 | 1,366 |
| **Dorsolateral prefrontal cortex** | 3,367 | 272 | 1,368 | 936 | 1,063 |
| **Ventrolateral prefrontal cortex** | 1,663 | 155 | 610 | 478 | 575 |
|  |  |  |  |  |  |
| **Sample names** | **Non-CpG total** | **Non-CpG promoter** | **Non-CpG exon** | **Non-CpG intron** | **Non-CpG intergenic** |
| **Angular gyrus** | 114,440 | 2,759 | 8,959 | 49816 | 55,665 |
| **Anterior caudate** | 185,634 | 4,594 | 13,697 | 79926 | 92,011 |
| **Cingulate gyrus** | 39,107 | 991 | 2,645 | 16057 | 20,405 |
| **Hippocampus middle** | 162,807 | 3,640 | 11,511 | 67620 | 83,676 |
| **Inferior temporal lobe** | 57,092 | 1,462 | 4,114 | 24275 | 28,703 |
| **Dorsolateral prefrontal cortex** | 57,290 | 1,304 | 4,043 | 23960 | 29,287 |
| **Ventrolateral prefrontal cortex** | 30,985 | 786 | 2,146 | 13651 | 15,188 |

Table S7 simple statistics of DMS counts by different genome features

| ID | Gene Name | Species | GOTERM_BP_DIRECT | GOTERM_CC_DIRECT | GOTERM_MF_DIRECT | KEGG_PATHWAY |
| --- | --- | --- | --- | --- | --- | --- |
| CXCR6 | C-X-C motif chemokine receptor 6(CXCR6) | Homo sapiens | GO:0006935~chemotaxis,GO:0006954~inflammatory response,GO:0007186~G-protein coupled receptor signaling pathway,GO:0019079~viral genome replication,GO:0070098~chemokine-mediated signaling pathway, | GO:0005886~plasma membrane,GO:0005887~integral component of plasma membrane,GO:0016021~integral component of membrane, | GO:0004930~G-protein coupled receptor activity,GO:0015026~coreceptor activity,GO:0016494~C-X-C chemokine receptor activity,GO:0019958~C-X-C chemokine binding, | hsa04060:Cytokine-cytokine receptor interaction,hsa04062:Chemokine signaling pathway, |
| CXCR6 | C-X-C motif chemokine receptor 6(CXCR6) | Macaca fascicularis | GO:0006935~chemotaxis,GO:0006954~inflammatory response, | GO:0005886~plasma membrane,GO:0016021~integral component of membrane, | GO:0015026~coreceptor activity,GO:0016494~C-X-C chemokine receptor activity, | mcf04060:Cytokine-cytokine receptor interaction,mcf04062:Chemokine signaling pathway, |
| AMDHD2 | amidohydrolase domain containing 2(AMDHD2) | Macaca fascicularis |  |  |  | mcf00520:Amino sugar and nucleotide sugar metabolism,mcf01130:Biosynthesis of antibiotics, |
| AMDHD2 | amidohydrolase domain containing 2(AMDHD2) | Homo sapiens | GO:0005975~carbohydrate metabolic process,GO:0006046~N-acetylglucosamine catabolic process,GO:0006048~UDP-N-acetylglucosamine biosynthetic process,GO:0019262~N-acetylneuraminate catabolic process, | GO:0005634~nucleus,GO:0005829~cytosol, | GO:0005515~protein binding,GO:0008448~N-acetylglucosamine-6-phosphate deacetylase activity,GO:0016810~hydrolase activity, acting on carbon-nitrogen (but not peptide) bonds,GO:0046872~metal ion binding, | hsa00520:Amino sugar and nucleotide sugar metabolism,hsa01130:Biosynthesis of antibiotics, |
| AATK | apoptosis associated tyrosine kinase(AATK) | Homo sapiens | GO:0006468~protein phosphorylation, | GO:0016021~integral component of membrane,GO:0048471~perinuclear region of cytoplasm, | GO:0004672~protein kinase activity,GO:0004674~protein serine/threonine kinase activity,GO:0005524~ATP binding, |  |
| CT62 | cancer/testis antigen 62(CT62) | Homo sapiens |  |  |  |  |
| MYADM | myeloid associated differentiation marker(MYADM) | Homo sapiens | GO:0001933~negative regulation of protein phosphorylation,GO:0010629~negative regulation of gene expression,GO:0010810~regulation of cell-substrate adhesion,GO:0030335~positive regulation of cell migration,GO:0030837~negative regulation of actin filament polymerization,GO:0031579~membrane raft organization,GO:0034115~negative regulation of heterotypic cell-cell adhesion,GO:0045217~cell-cell junction maintenance,GO:0061028~establishment of endothelial barrier,GO:0072661~protein targeting to plasma membrane,GO:0090038~negative regulation of protein kinase C signaling,GO:1900026~positive regulation of substrate adhesion-dependent cell spreading, | GO:0001726~ruffle,GO:0005886~plasma membrane,GO:0005911~cell-cell junction,GO:0016021~integral component of membrane,GO:0030864~cortical actin cytoskeleton,GO:0045121~membrane raft,GO:0070062~extracellular exosome, |  |  |
| MYADM | myeloid associated differentiation marker(MYADM) | Macaca fascicularis |  | GO:0016021~integral component of membrane, |  |  |
| LOC102132555 | olfactory receptor 2T8(LOC102132555) | Macaca fascicularis |  |  |  | mcf04740:Olfactory transduction, |
| TP53I3 | tumor protein p53 inducible protein 3(TP53I3) | Homo sapiens | GO:0006739~NADP metabolic process,GO:0042981~regulation of apoptotic process,GO:0055114~oxidation-reduction process, | GO:0005829~cytosol,GO:0070062~extracellular exosome, | GO:0003960~NADPH:quinone reductase activity,GO:0008270~zinc ion binding,GO:0016491~oxidoreductase activity,GO:0042803~protein homodimerization activity,GO:0048038~quinone binding,GO:0070402~NADPH binding, | hsa04115:p53 signaling pathway, |
| TP53I3 | tumor protein p53 inducible protein 3(TP53I3) | Macaca fascicularis |  |  |  | mcf04115:p53 signaling pathway, |
|  |  |  |  |  |  |  |

Table S8 DAVID annotation and GO terms from 11 DMGs

|  |  |  |  |  |  |
| --- | --- | --- | --- | --- | --- |
| Category |  |  |  |  | % of genome |
| total repeat elements |  | count |  | 5,174,162 |  |
|  |  | size |  | 1,405,083,609 | 48.93% |
| LTR |  | count |  | 677,047 |  |
|  |  | size |  | 246,238,124 | 8.57% |
| LINE |  | count |  | 1,435,110 |  |
|  |  | size |  | 586,457,504 | 20.42% |
| SINE |  | count |  | 1,754,105 |  |
|  |  | size |  | 387,560,307 | 13.50% |
| other repeat elements |  | count |  | 1,307,900 |  |
|  |  | size |  | 184,827,674 | 6.44% |

Table S9. Summary statistics of repeat elements


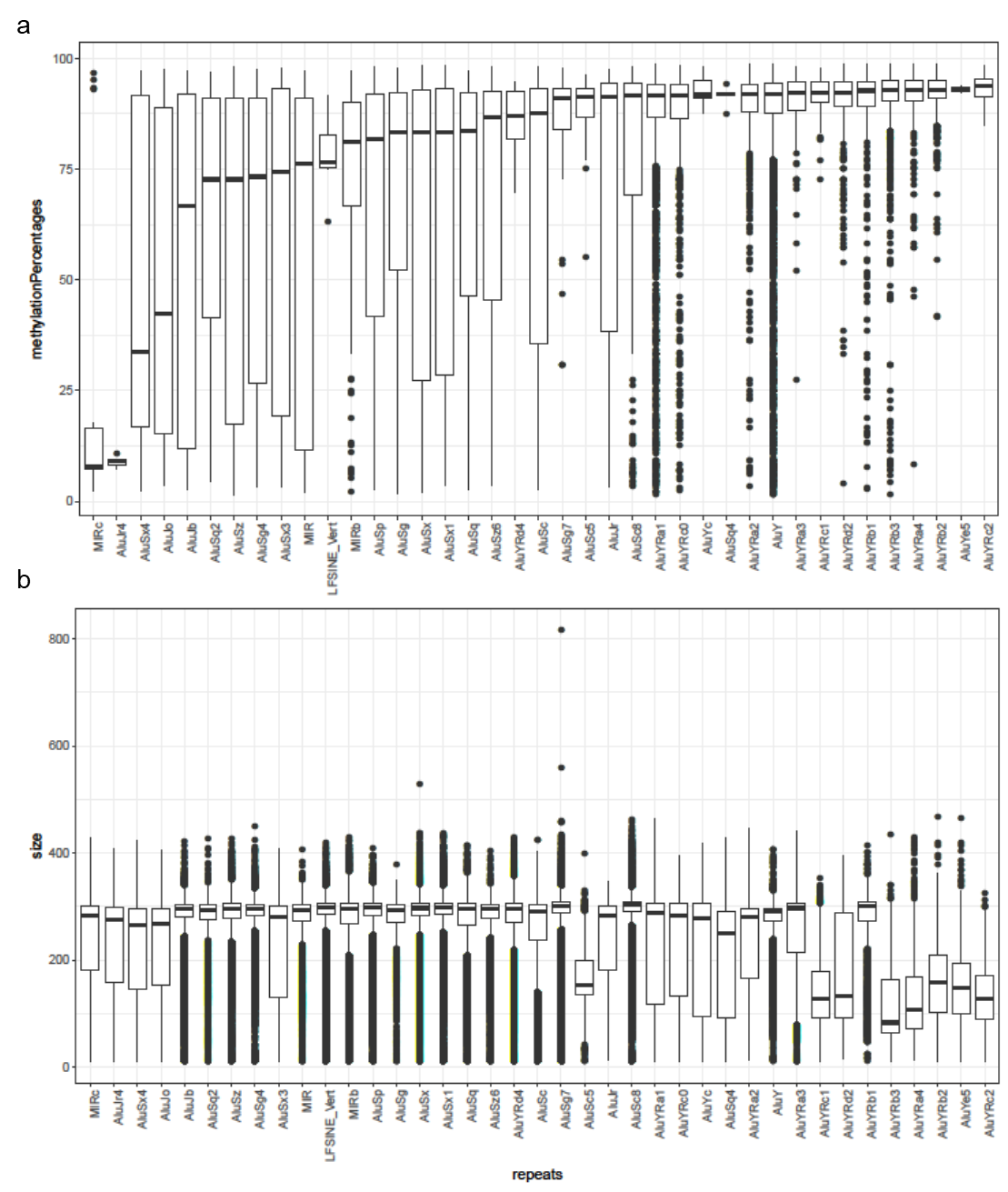


Figure S1. DNA methylation status and size of SINEs. a. Box plot of DNA methylation of SINEs, by repeat type. b. Box plot of size of each type of SINE.
